# Case report: Abdominal hernia repair using a surgical wire and an autologous omental graft in a Japanese Black calf

**DOI:** 10.3389/fvets.2023.1119034

**Published:** 2023-01-26

**Authors:** Reiichiro Sato, Sueun Kim, Shoichi Okada, Tomonobu Ikedo, Hiroyuki Satoh, Adrian Steiner

**Affiliations:** ^1^Graduate School of Medicine and Veterinary Medicine, University of Miyazaki, Miyazaki, Japan; ^2^Kusu Livestock Hygiene Service Center, Oita Prefectural Government, Oita, Japan; ^3^Vetsuisse Faculty, Clinic for Ruminants, University of Bern, Bern, Switzerland

**Keywords:** abdominal hernia, abdominal wall reconstruction, autologous omental graft, calf, spondylocostal dysostosis, wire

## Abstract

**Background:**

Hernia formation is a well-known abdominal wall disorder in calves and most often occurs in the umbilical region. In addition, it occurs in the abdominal wall outside the umbilical region. It has been reported to involve acquired factors, such as external force to the lower or lateral abdominal wall, trauma, muscle weakness, and, although rare, congenital or hereditary factors. Although there have been reports on the repair of abdominal wall hernias caused by abnormal muscle formation, there have been no reports on the treatment of abdominal wall hernias caused by abnormal vertebral and rib formation in cattle or other ruminants. In this study, for the first time, we describe a case of successful closure of a hernia in the left flank caused by malformation of the vertebrae and ribs. The repair was performed by narrowing the ribs using a surgical wire and covering the defect with a free omental graft.

**Case presentation:**

A male Japanese Black calf showed a mild bulge of the left abdominal wall and abnormal morphology of the left ribs immediately after birth. At 3 months of age, computed tomography revealed fusion of the 9th, 10th, and 11th thoracic vertebrae and missing formation of the 10th and 11th left ribs at the thoracic vertebral fusion site. Additionally, a 15.2 × 24.4 cm abdominal hernia had formed in the abdominal wall. During surgery, the ribs forming the hernia were narrowed with a surgical wire, and the hernia was covered with a large autologous free omental graft to prevent intra-abdominal organ prolapse. At the monthly follow-up (11 months after surgery), the calf had developed similarly to other calves of the same age on the farm, and no complications were noted.

**Conclusions:**

The current case shows that a combination of rib correction using a surgical wire combined with transplantation of a free autologous greater omentum graft was shown to be a possible treatment option for surgical repair of large abdominal wall hernias caused by rib malformation.

## 1. Introduction

Hernia formation is one of the most common abdominal disorders observed in calves. It most commonly occurs in the umbilical region (1–4) and rarely at other sites of the abdominal wall (1, 5–7). Abdominal wall hernias are commonly caused by acquired factors, such as external force to the lower or lateral abdominal wall, trauma, or muscle weakening (1, 7, 8). However, congenital or hereditary factors due to abnormal muscle formation have also been identified (5, 6, 9).

Abdominal wall hernias are easily diagnosed based on the characteristic clinical appearance of the abdominal wall. Palpation of the hernial site can be used to assess the size of the hernial foramen, reducibility, and presence of complications, such as abscesses (5). Hernia content can be evaluated accurately by ultrasonography (10).

Surgical hernia repair is dependent on the age of the animal and the location and size of the hernia, and correction can be performed by suture closure or mesh repair (7). If the diameter of the hernial ring is small, the method of choice is a single interrupted suture or a modified mayo mattress (vest-over-pants) suture repair (11). If the hernial ring is large, the method of choice is repair using a mesh of absorbable, nonabsorbable, or semi-absorbable material (2, 7, 12). Among hernias, abdominal wall hernias are suitable for mesh repair because the abdominal wall at the site of the defect is often fragile, and suture-based repair may induce muscle tears due to excessive tension on the sutures (8).

This case report presented an abdominal wall hernia caused by spondylocostal dysostosis (SCDO) of the vertebrae and ribs. Although there have been reports on the repair of abdominal wall hernias caused by dysplastic muscles associated with the prune belly-like syndrome (6, 9, 13), there are no reports on abdominal wall hernias caused by SCDO in cattle.

This report aims to demonstrate that, for large abdominal wall hernias caused by rib malformations, such as SCDO, combining surgical wiring and transplantation of an autologous free omental graft is effective in closing a hernial opening of considerable size.

## 2. Case description and diagnostic assessment

A male Japanese Black calf showed a slight bulge on its left abdominal wall and abnormal morphology of the left ribs immediately after birth. The calf's left abdominal wall bulge gradually increased in size with growth, but there was no abnormality in the calf's general condition and food and water intake. Moreover, no symptoms of gastrointestinal disease were observed; thus, the animal was kept under normal management conditions.

At the age of 3 months, with a body weight of 114 kg, the calf was admitted to Miyazaki University Veterinary Teaching Hospital for diagnostic and prognostic evaluation and treatment. On initial examination, the calf's heart rate, respiratory rate, and rectal temperature were 110 beats/min, 36 breaths/min, and 39.4°C, respectively.

Appetite, urination, and defecation were physiological, and no signs of neurological disease were noted. The calf's complete blood count was within normal limits (white blood cell count, 9,500 cells/μL; red blood cell count, 1,090 × 104 cells/μL; and thrombocytes, 39.5 × 104 cells/μL). The calf's serum examination showed no abnormalities (sodium, 144 [normal reference value, 132–152] mmol/L; potassium, 4.8 [normal reference value, 3.9–5.8] mmol/L; chloride, 100 [normal reference value, 97–111] mmol/L) (14).

**Figure 1:**
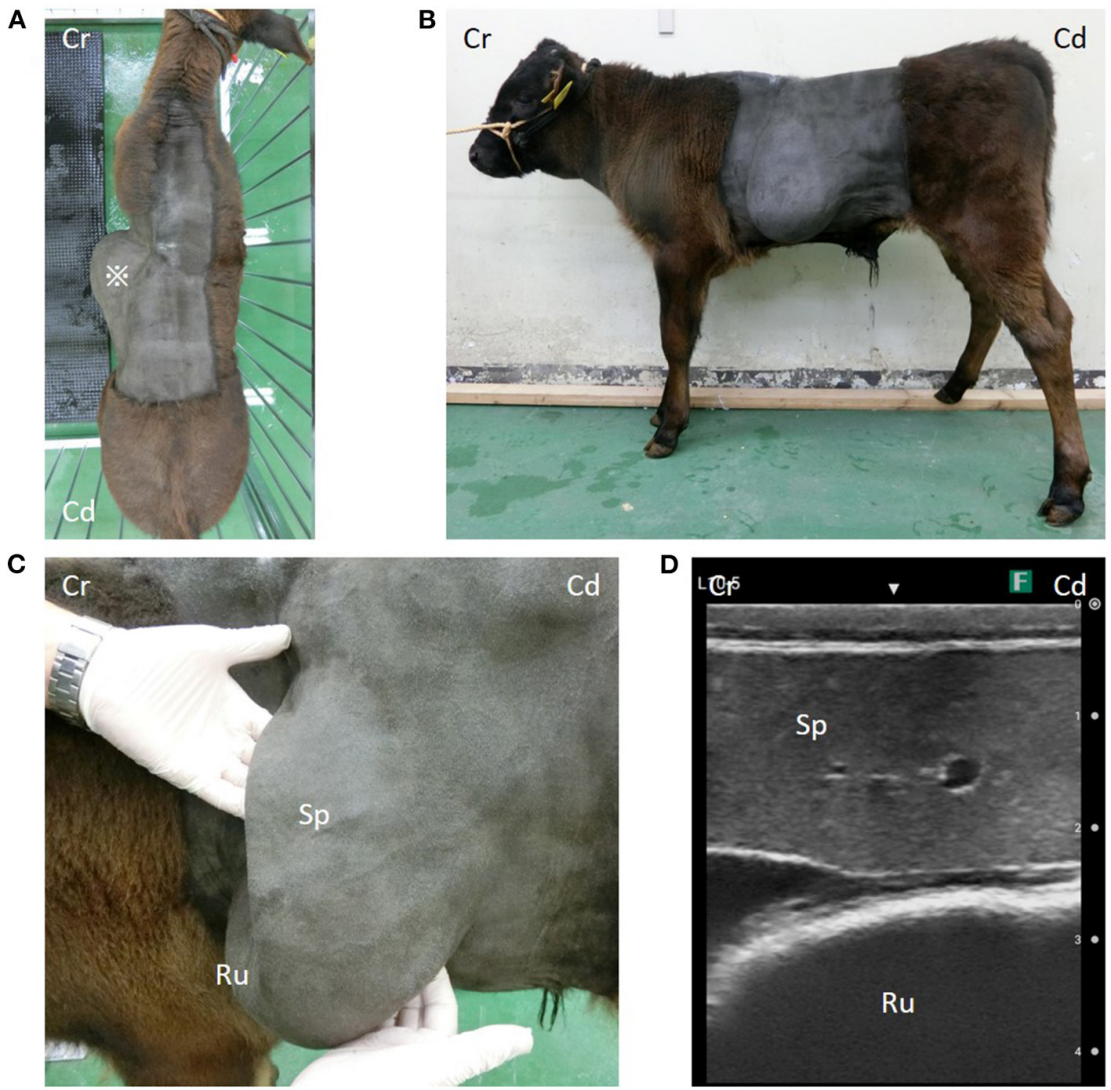
**(A)** *A large bulge on the left side of the abdominal wall. **(B)** A cystic organ is considered to be the rumen, and an organ is considered to be the spleen prolapse through the hernial hole. **(C)** Organs in the hernial sac and **(D)** their ultrasound images. Cr, Cranial; Cd, Caudal; Sp, spleen; Ru, rumen.

In dorsal and left lateral views, the calf's left lateral abdominal wall was markedly distended (Figures 1A, B). Palpation of the abdominal bulge revealed a large curvature of the left last rib caudally and a large curvature of a rib cranially, forming a large hernial ring. On ultrasonography (7.0–8.0-MHz variable linear probe, iViz air, FUJIFILM, Tokyo, Japan), the spleen and dorsal ruminal sac protruded through the hernial ring into the hernial sac (Figures 1C, D). These organs could easily be pushed back manually into the abdominal cavity. Evidence of coverage of the hernial ring with musculature was not observed.

**Figure 2:**
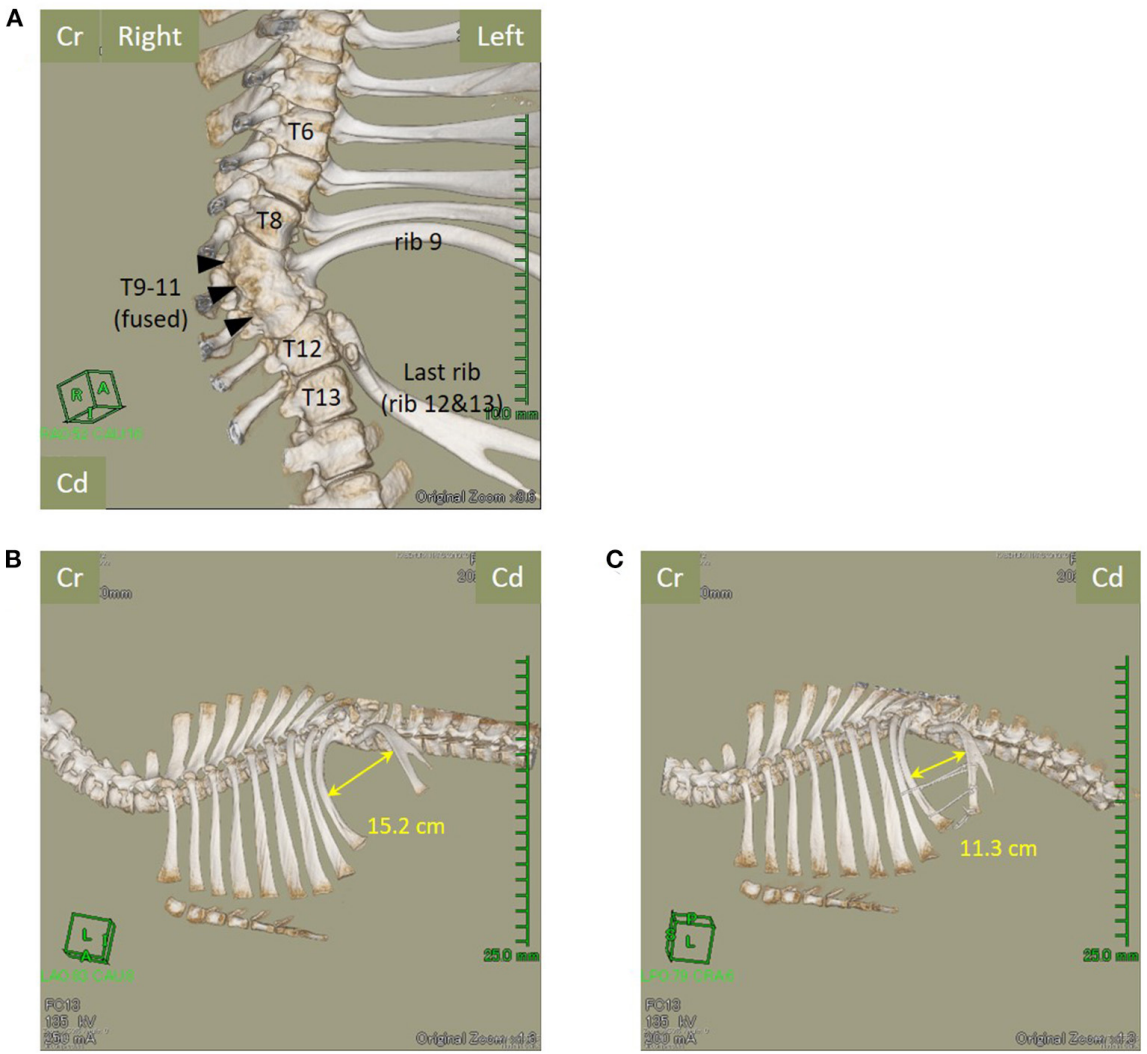
The fusion of the 9th, 10th, and 11th thoracic vertebrae (arrowheads) and loss of the left-sided rib, with the right-sided rib being partially erased (ventral view). **(A)** The 11 left-sided ribs, the last rib (12th and 13th ribs), and the maximum intercostal width of the hernial foramen reduce from 15.2 cm **(B)** before surgery to 11.3 cm **(C)** after surgery. Cr, Cranial; Cd, Caudal; T, Thoracic vertebra.

The calf was sedated with intravenous xylazine hydrochloride (0.2 mg/kg) and underwent computed tomography (CT) using a helical 16-row multi-detector CT device (Aquilion Lightning TSX-035A, Canon, Ohtawara, Japan) for the preoperative evaluation of skeletal abnormalities. CT revealed the presence of 11 left ribs (ribs 12 and 13 were considered partially fused). In addition, the 9th, 10th, and 11th thoracic vertebrae appeared completely fused and curving leftward (Figure 2A). The ribs 10 and 11 were missing, with the rib at the thoracic fusion site (rib 9) curving cranially and the last rib (ribs 12 and 13) curving caudally, forming a hernial ring. The maximum intercostal width was 15.2 cm (Figure 2B). There was no evidence of anatomical abnormalities of the sternum.

## 3. Surgical intervention

With continuing growth, we expected the hernial opening to increase in size and the organ prolapse to cause clinical problems. Therefore, we planned hernia repair surgery to correct the enlarged intercostal space caused by the deformed ribs and to permanently position the prolapsed organs back into the abdominal cavity by closing the hernial opening.

Because the hernial ring was cranially and caudally made of ribs and sufficient muscular and fibrous tissue was not present, the hernia was closed by tightening the intercostal space using a surgical wire and closing the remaining hernial opening with a free autologous omental graft. The surgical technique comprised the following three stages: [1] dissection of the greater omentum using a right lateral abdominal approach, [2] tightening of the intercostal space using a surgical wire, and [3] transplantation of the dissected free autologous omental graft into the hernia foramen.

The calf was fasted for 12 h before surgery. Then, it received an intravenous injection of cefazolin sodium (5 mg/kg) (Cefazolin-Chu; Fujita Pharmaceutical, Tokyo, Japan) to prevent perioperative infection and flunixin meglumine (2 mg/kg, Forvet50, MSD, Tokyo, Japan) for pain relief.

Subsequently, the calf was sedated with intravenous xylazine hydrochloride (0.2 mg/kg) and placed in left lateral recumbency. Next, general anesthesia was induced by continuous administration of isoflurane (Isoflu; Zoetis Japan, Tokyo, Japan) at a concentration of 2% in oxygen. Local anesthesia with procaine hydrochloride (Adsan; Riken Vets Pharma, Saitama, Japan) was administered subcutaneously around the incision line of the right flank.

A 20-cm incision was performed in the center of the right paralumbar fossa, starting approximately 5 cm ventral to the transverse processes of the lumbar vertebrae. After the abdomen was opened, the greater omentum of the outer sheet covering the intestinal tract was pulled out of the incision site, and a 30 × 30-cm free omental graft was collected. The harvested omental graft was temporarily stored in saline-soaked gauze and refrigerated until use. The defect in the greater omentum was closed with a simple continuous suture using polyglactin 910, synthetic absorbable multifilament suture material (USP 0, Vicryl, Johnson & Johnson, Tokyo, Japan). The peritoneum and transverse abdominal muscle were closed using synthetic, absorbable, multifilament polyglycolic acid suture material (USP 3+4, Opepolyx, Alfresa Pharma, Osaka, Japan) in a continuous suture pattern. The internal and external oblique abdominal muscles were separately sutured with simple continuous sutures using polyglycolic acid synthetic absorbable material (USP 3+4, Opepolyx). The skin incision was closed with intradermal buried sutures using polyglactin 910, synthetic absorbable multifilament suture material (USP 0, Vicryl, Johnson & Johnson, Tokyo, Japan).

Next, the calf was placed in right lateral recumbency. The left abdominal wall was incised through a straight skin incision approximately 30 cm in length in a diagonal craniodorsal to caudoventral direction centered over the hernial foramen. At incision, there was no musculature, and only fat and the peritoneum were observed under the skin of the hernial sac. The hernial foramen was approximately 15 × 25 cm in size, confirming the preoperative findings, and the rumen and spleen were clearly identified on palpation.

**Figure 3:**
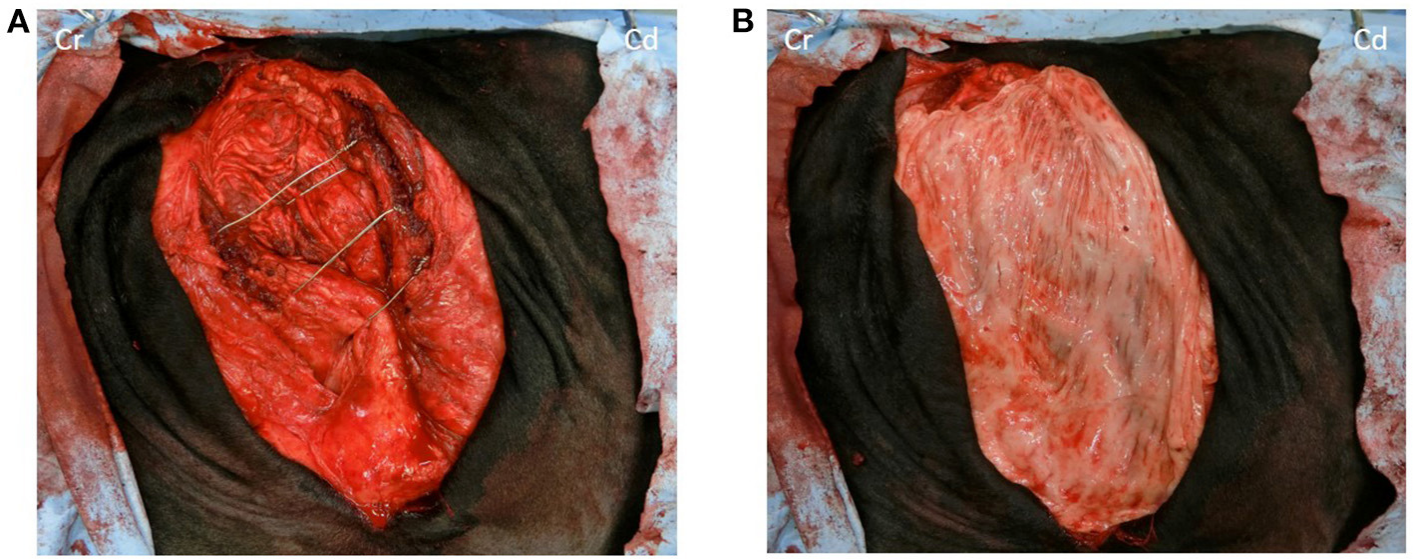
**(A)** No musculature is observed under the skin of the hernia foramen, only white peritoneum; ventral two-thirds of the last rib and between the seventh and eighth ribs and ventral one-sixth of the last rib and the ventral end of the last rib is wired to the eighth and ninth rib to reduce the intercostal space as much as possible. **(B)** The hernia rings are covered with a large autogenous-free graft. Cr, Cranial; Cd, Caudal.

A surgical 18-gauge wire was used to narrow the fused last two ribs and the ninth rib to tighten the intercostal space as much as possible (Figure 3A). Subsequently, the hernial ring was covered with the omental graft preserved in saline. Additionally, a simple continuous suture was made using multifilament polyglycolic acid suture material (USP 3+4, Opepolyx, Alfresa Pharma, Osaka, Japan) (Figure 3B). The skin incision was closed as described for the incision in the right flank.

**Figure 4:**
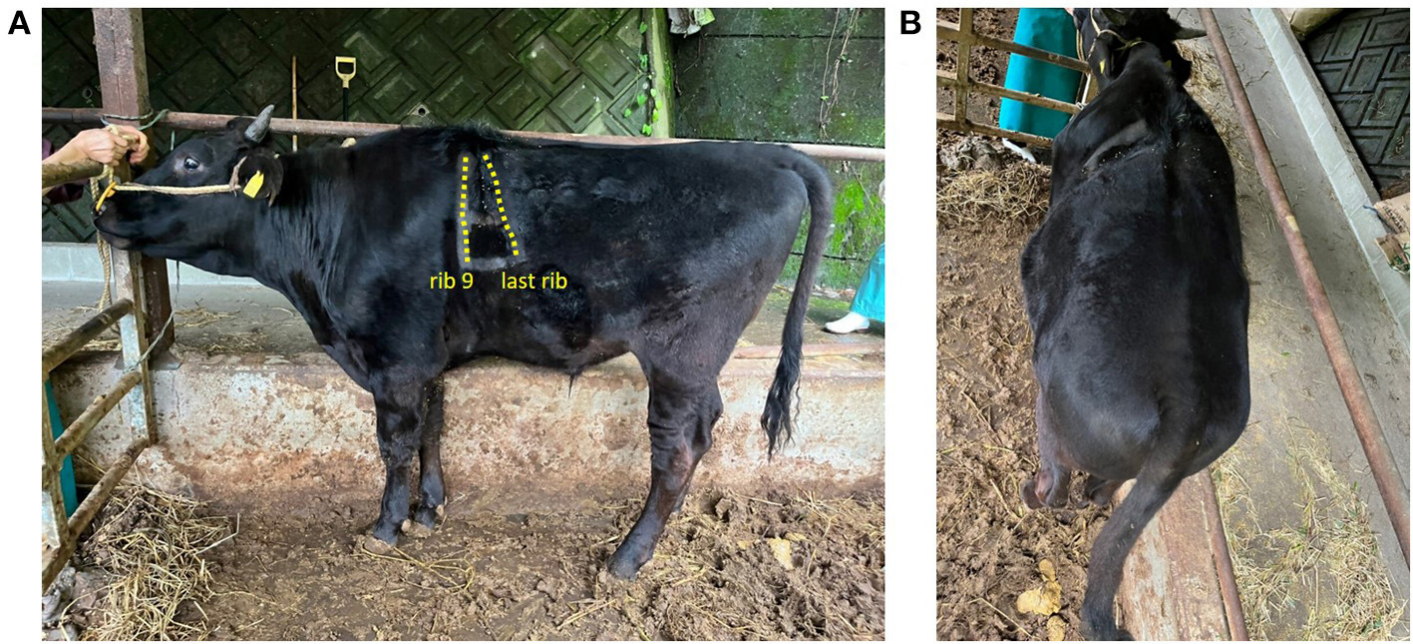
Eleven months postoperatively calf's age was 14 months. The dashed line area is the rib. The cranial dashed line indicates the 9th rib, and the caudal line indicates the last rib. **(A)** Left-sided and **(B)** dorsal areas.

Immediately after surgery, control CT revealed that the maximum intercostal width between the 9th and 12th/13th ribs had decreased from 15.2 cm to 11.3 cm (Figure 2C). For the initial three postoperative days, flunixin meglumine (2 mg/kg, Forvet50, MSD, Tokyo, Japan) was administered intravenously once daily, and cefazolin sodium (5 mg/kg) was administered intravenously once daily for 10 days. For the initial three postoperative days, feed was restricted to approximately half the normal amount to prevent rapid dilation of the rumen. At 48 h postoperatively, ruminal motility returned to normal. The patient's postoperative development was good, and the calf was discharged on postoperative day 10. Follow-up examination at the farm was performed at postoperative month 11. The calf had developed similar to its herd mates on the farm, without evidence of symptoms of gastrointestinal disorders or complications at the surgical site (Figure 4).

## 4. Discussion

Although there have been reports of treatment of abdominal wall hernias in cattle due to thin abdominal muscular layers (6, 9, 13), there have not been any reports of cases caused by thoracic spine and rib abnormalities, as in the current case, or of surgical treatment and follow-up of such cases.

The most serious complication of abdominal wall hernias is the incarceration of abdominal hollow organs, causing ischemia, luminal obstruction, and pain (15–17). However, this likely occurs when the hernial defect is of similar diameter as the intestines and the hernial sac is large (17).

Fortunately, in this case report, the hernia was in the left abdominal wall, and the organs prolapsed were the rumen and spleen. Moreover, incarceration did not occur. Thus, the general condition of the calf was normal. However, the calf was expected to develop gastrointestinal symptoms as the rumen volume and weight increased with growth.

If the hernial ring is large, an artificial mesh is inserted into the hernial ring. However, there is an increased risk of infection, especially with mesh made of a nonabsorbable material (18). In addition, absorbable materials may not retain tensile strength, and recurrence may occur.

Autologous tissue transplantation has been performed to prevent large hernias in the chest and after rib resection. For autologous muscle flaps in the chest, the vastus lateralis muscle is sometimes used in terms of position, size, and vascular supply (19). In contrast, the greater omentum is used in various surgeries, from general to gynecological, as it is excellent for revascularization and is immunologically viable outside of muscle tissues (20–22).

Therefore, in the present study, the intercostal space forming the hernial ring was tightened by narrowing the involved ribs with surgical wires to prevent further expansion with growth, and the autologous omental graft was used as the material covering the hernial opening.

There are two types of autologous tissue flaps used in surgery: pedicle and free flaps. The greater omentum pedicle flap is transferred to any site within the abdominal cavity and outside the abdominal wall using the greater omentum extension method (23). However, the greater omentum pedicle flap has a limited reachable area; thus, greater omentum-free flaps are used to cover defects in extremity wounds (21, 24). When a greater omentum-free flap is used, an end-to-end or end-to-side microvascular anastomosis is often performed between the artery at the site of coverage and the omental artery (24). However, there have been cases of successful wound coverage and reconstruction without anastomosis (24). A free omental graft was also used in the current case. Because of time constraints, we decided against performing arterial anastomosis.

Eleven months after the operation, no gastrointestinal symptoms or adverse effects on the calf's growth were observed; however, continuous careful monitoring is indicated for early detection of complications associated with tightening the hernial ring with a surgical wire.

In conclusion, the current case shows that for surgical repair of large abdominal wall hernias caused by disorders, such as SCDO, a combination of rib correction using a surgical wire combined with transplantation of a free autologous greater omentum graft was shown to be a possible treatment option.

## 5. Patient perspective

This study was conducted according to the owner's agreement for the advanced, informed written consent. Furthermore, we obtained written informed consent from the owner for the use of information and clinical data of the present case in a publication.

## Data availability statement

The original contributions presented in the study are included in the article/supplementary material, further inquiries can be directed to the corresponding author.

## Ethics statement

Ethical review and approval was not required for the animal study because the case report is a description of a clinical case. Written informed consent was obtained from the owners for the participation of their animals in this study.

## Author contributions

RS and SK performed surgical therapy, reviewed the literature, were responsible for data collection, and prepared the manuscript. HS performed the clinical and CT examinations. SO and TI supported the clinical examination and data collection. AS edited the manuscript and supervised the study. All authors have read and approved the final manuscript, contributed to the manuscript, and approved the final version.

## References

[1] Oehme FW. Lateral abdominal hernia in a cow. Cornell Vet. (1965) 55:321–9. 14317490

[2] Wintzer HJ. Methods for surgical treatment of bovine abdominal hernias. J Am Vet Med Assoc. (1962) 141:131–4. 14007626

[3] Smith BP. Large Animal Internal Medicine, 3rd ed. St. Louis, MO: Mosby-Elsevier Publishing (2009). p. 289–651.

[4] Fesseha H. Umbilical hernia in cross Holstein Friesian calf and its surgical management: a case report. Vet Med. (2020) 5:39–42. 10.17140/VMOJ-5-147

[5] Weaver D Steiner A St Jean G. Abdominal Surgery. In:Weaver D, editors. Bovine Surgery and Lameness. Oxford: Blackwell Publishing (2005). p. 75-139. 10.1002/9780470751138

[6] Sala G Boccardo A Coppoletta E Riccaboni P Zani DD Pravettoni D. Prune belly-like syndrome in two calves. Large Anim Rev. (2018) 24:201–4.

[7] Amare E Haben F. Hernias in farm animals and its management technique-a review. IJCMCR. (2020) 4:001. 10.46998/IJCMCR.2020.04.000091

[8] Astiz BS González MJV Elvira PL Rodriguez BA Camon UJ. Primer caso de síndrome prune belly-like en una ternera de raza holstein con quistes hepáticos serosos. Rev Electron Vet. (2007) 8:1–8.

[9] Steiner A Lejeune B. Ultrasonographic assessment of umbilical disorders. Vet Clin North Am Food Anim Pract. (2009) 25:781–94. 10.1016/j.cvfa.2009.07.012 19825444

[10] Spadola F Neve VC Costa GL Musicò M Spadaro A Antoci F. Surgical approach and etiopathogenetic considerations to the umbilical tumefactions in cattle: case review in twenty years (2000/2020). Vet Anim Sci. (2022) 17:100258. 10.1016/j.vas.2022.100258 35784586 PMC9241135

[11] Rings DM. Umbilical hernias, umbilical abscess, and urachal fistulas. Surgical considerations. Vet Clin North Am Pract. (1995) 11:137–48. 10.1016/S0749-0720(15)30512-0 7796328

[12] Edwards GB. Abdominal cavity. In:Mair TS Love S Schumacher J Smith RKW Frazer G, editors. Equine Med Surg Reproduction. Elsevier: Saunders Ltd (2012). p. 67–75. 10.1016/B978-0-7020-2801-4.00004-3

[13] Ando Y Tsuka T Okamoto Y. Lateral abdominal hernia associated with thin abdominal musculature in a calf. J Vet Med Sci. (2022) 84:533–7. 10.1292/jvms.21-0653 35197414 PMC9096036

[14] Kaneko JJ Harvey JW Bruss ML. Blood Analyte Reference Values in Large Animals: Clinical Biochemistry of Domestic Animals. 6th ed. Elsevier: Oxford. (2008). p. 882–8.

[15] Mair TS Love S Schumacher J Smith RKW Frazer G. Equine Medicine, Surgery and Reproduction-E-Book. Amsterdam : Elsevier Health Sciences. (2013).

[16] Fossum TW. Small Animal Surgery 5th ed. Amsterdam : Elsevier Health Sciences (2018). p. 518–22.

[17] Smeak DD. Abdominal wall reconstruction and hernias. In: Johnston SA Tobias KM. Veterinary Surgery Small Animal 2nd ed. Elsevier Missouri (2018). p. 1564–70.

[18] Limpert JN Desai AR Kumpf AL Fallucco MA Aridge DL. Repair of abdominal wall defects with bovine pericardium. Am J Surg. (2009) 198:e60–5. 10.1016/j.amjsurg.2009.01.027 19811771

[19] Liptak JM Dernell WS Rizzo SA Monteith GJ Kamstock DA Withrow SJ. Reconstruction of chest wall defects after rib tumor resection: a comparison of autogenous, prosthetic, and composite techniques in 44 dogs. Vet Surg. (2008) 37:479–87. 10.1111/j.1532-950X.2008.00413.x 18986316

[20] Williams R White H. The greater omentum: its applicability to cancer surgery and cancer therapy. Curr Probl Surg. (1986) 23:789–865. 10.1016/0011-3840(86)90007-9 3780294

[21] Mazzaferro D Song P Massand S Mirmanesh M Jaiswal R Pu LLQ. The omental free flap—a review of usage and physiology. J Reconstr Microsurg. (2018) 34:151–69. 10.1055/s-0037-1608008 29141258

[22] Di Nicola V. Omentum a powerful biological source in regenerative surgery. Regen Ther. (2019) 11:182–91. 10.1016/j.reth.2019.07.008 31453273 PMC6700267

[23] Ross WE Pardo AD. Evaluation of an omental pedicle extension technique in the dog. Vet Surg. (1993) 22:37–43. 10.1111/j.1532-950X.1993.tb00366.x 8488673

[24] Maloney CT Jr Wages D Upton J Lee WP. Free omental tissue transfer for extremity coverage and revascularization. Plast Reconstr Surg. (2003) 111:1899–904. 10.1097/01.PRS.0000056874.31920.7D 12711950

